# Targeting indigenous *Schizosaccharomyces japonicus* for genotype exploration and organic acid degradation analysis

**DOI:** 10.3389/fmicb.2025.1569585

**Published:** 2025-06-04

**Authors:** Qiling Chen, Chunxiao Wang, Zhanyan Zhang, Zhangyu Yang, Wenya Zhou, Jiaojiao He, Angxin Song, Yuangen Wu, Shuyi Qiu, Lizhen Han

**Affiliations:** ^1^Key Laboratory of Plant Resource Conservation and Germplasm Innovation in Mountainous Region (Ministry of Education), College of Life Sciences/Institute of Agro-Bioengineering, Guizhou University, Guiyang, China; ^2^School of Liquor and Food Engineering, Guizhou University, Guiyang, China

**Keywords:** non-*Saccharomyces*, malic acid, microsatellite, fermentation, deacidification

## Abstract

*Schizosaccharomyces japonicus* belongs to fermentative non-*Saccharomyces* yeast, which has been reported to be predominant in the late stage of natural wine fermentation, and can coexist with *S. cerevisiae*. This study targeted the indigenous *S. japonicus* strains found in Guizhou, China, to explore their genotype diversity, and to further analyze their fermentation properties especially on the degradation capacity of malic acid. Seven microsatellite loci were developed including GA1, CG3, SyGAA2, C11, SaGAA1, C12, and TG2, and 43 genotypes were identified from 63 *S. japonicus* strains. All *S. japonicus* strains showed high malic acid reduction rate (higher than 90%) by initial metabolic analysis, except FBKL2.9SZJ-43 showing medium malic acid reduction rate (89.47%). The highest malic acid reduction rate was found in FBKL2.9SZJ-37 (98.64%). Ten *S. japonicus* strains were selected for further simulated fermentations based on their genotype, flocculation, and organic acid degradation traits. Among them, FBKL2.9SZJ-37 and FBKL2.9SZJ-55 showed good fermentation ability, strong malic acid degradation capacity, and relative weak tartaric acid degradation performance under both simulated fermentation conditions. Further fermentations using *Rosa sterilis*, *Rosa roxbunghii* Tratt *juice*, and *Cabernet Sauvignon*, verified the fermentation characteristics of the two *S. japonicus* strains when compared to *S. cerevisiae*. Especially for FBKL2.9SZJ-37, it showed good ethanol production, stronger glycerol formation and acid degradation ability, with malic acid reduction rate being 60.50, 64.98 and 98.32% in fermentations of three fruits, respectively. This study displays initial explorations on genotyping and fermentation application of indigenous *S. japonicus* especially for malic acid type fruit wine making, which would help to the development and application of potential excellent non-*Saccharomyces* yeast sources.

## 1 Introduction

The types and contents of organic acids differ in different fruits and affect the taste of fruit wine. Oxalic acid, tartaric acid, malic acid, acetic acid, citric acid, succinic acid and lactic acid are the most common seven organic acids in fruit wine (Chen et al., 2024). *Cabernet Sauvignon* is a common grape variety for making red wine, which is native to the Bordeaux region of France. It is widely planted in China, and in some cool wine regions it owns high organic acids due to the harsh weather in mature season. *Cabernet Sauvignon* belongs to tartaric acid type fruit with total organic acids of 16.14 g/kg. The main organic acids in it are tartaric acid and malic acid (Yang et al., 2016). The taste of sour is regarded as one of the most important sensory attributes of a quality wine, which is mainly brought about by the mixture of organic acids transferred from the grape flesh to the wine during the winemaking process (Picariello et al., 2019). In addition to grape, there are many unique fruit resources distributing in different ecological environments of China. For example, *Rosa roxbunghii* Tratt and *Rosa sterilis* are a unique rose family fruit resource in Guizhou province, which are regarded to be richest in vitamin C of all fruits (Shan et al., 2024; Ding and Sun, 2024). Both *Rosa roxbunghii* Tratt and *Rosa sterilis* belong to malic acid type fruit with main organic acid being malic acid. And both fruits were reported to contain higher organic acid content than *Cabernet Sauvignon*, with *Rosa sterilis* being 72.30 g/L and *Rosa roxbunghii* Tratt being 44.54 g/kg (An et al., 2011; Chen et al., 2024). High levels of malic acid can bring a harsh and sharp sour taste, and malic acid is more likely to be metabolized by bacteria than other organic acids. Therefore, deacidification, especially reducing malic acid, is conducive to improving the taste quality and microbial stability of fruit wine.

Microbial deacidification is a common and natural way in fruit wine processing, mainly for degrading malic acid (Cao et al., 2023). Lactic acid bacteria are the common acid-reducing microorganisms, which can transform malic acid to lactic acid by malolactic fermentation. The malolactic fermentation is catalyzed by malolactase, and achieve the purpose of acid reduction by the transformation of dicarboxylic malic acid to monocarboxylic lactic acid. *Schizosaccharomyces* has long been concerned with the degradation of organic acids (Volschenk et al., 2003; Benito et al., 2016). *S. pombe* can metabolize malic acid by malo-alcoholic fermentation, transforming malic acid to ethanol and carbon dioxide (Domizio et al., 2018; Wang et al., 2023). Further study on the acid-reducing capacity of *Schizosaccharomyces* is meaningful for fruit wine processing, because the metabolism happens during alcoholic fermentation, which can reduce the process complexity.

The genus of *Schizosaccharomyces* is the most typical biological model of cell division and reproduction, which has been widely studied in cell biology. Previous researches have reported abundant *Schizosaccharomyces* resources in Guizhou (Wang et al., 2019), and the positive impact of *Schizosaccharomyces* yeast on wine aroma, taste and color stability (Portaro et al., 2022; Wang et al., 2022). However, few studies have been performed on intraspecific diversity of non-*Saccharomyces* yeast, which is unhelpful for the development and application of potential excellent non-*Saccharomyces* yeast. Microsatellite analysis has displayed excellent intraspecific typing effect, due to its stability and polymorphism (Franco-Duarte et al., 2009; Pfliegler et al., 2014), which can be used to analyze the genetic diversity of a large number of strains (Masneuf-Pomarede et al., 2016), and to define and describe the relationship among strains associated with their geographical or technical origins (Schuller and Casal, 2007). Microsatellite analysis has only been applied to five non-*Saccharomyces* yeast species for diversity investigation, including *Hanseniaspora uvarum*, *Lachancea thermotolerans*, *Methchinikowia guilliermondii*, *Starmerella bacillaris* and *Torulaspora delbrueckii* (Wang et al., 2023). Although the genome reports of non-*Saccharomyces* yeast have increased, there are still a lot of data unavailable for applying microsatellite analysis compared to *S. cerevisiae* (Masneuf-Pomarede et al., 2016). The development of microsatellite loci in *Schizosaccharomyces* can provide theoretical basis for their genetic diversity analysis, and reliable molecular basis for monitoring different strains during wine fermentation.

The *S. japonicus* strains used in this study based on our previous investigation on indigenous yeast in crystal grape (unpublished data), which found *S. japonicus* being main yeast species during middle and late stages of spontaneous fermentations of crystal grapes collected from different countries in Guizhou, China. The aim of this study is to develop new microsatellite loci for *S. japonicus* genotype analysis, to screen excellent strains with strong malic acid reduction ability while relatively weak tartaric acid degradation capacity, to evaluate the fermentation capacity of the screened strains by simulated fermentations, and finally to verify its deacidification and fermentation potential in different fruit wine fermentations.

## 2 Materials and methods

### 2.1 Yeast strains used in this study

This study targeted 63 *S. japonicus* isolates, which were collected from spontaneous fermentation of crystal grape in 2020 from three sources in Guizhou province, with 22 isolates from S7 (Sandu county in July), 14 isolates from S8 (Sandu county in August), and 27 isolates from X9 (Xifeng county in September). All the isolates were identified by a combination of WL medium, 5.8S-ITS-RFLP, and 26S rRNA gene D1/D2 sequencing analysis, with DNA extraction and PCR amplification as described by Wang et al. (2019). Their colonies on WL identification medium were characterized by green colonies with a tray, their electrophoretic profiles of 5.8S-ITS-RFLP were 550 bp + 150 bp, and the sequence of D1/D2 domain of 16S rRNA gene of representative strains were numbered OP364837-OP364843 at NCBI. All the isolates were kept in fermentation engineering and biopharmaceutical province key laboratory. The strain FBKL2.9SZJ-29 was also stored in China Center for Type Culture Collection with number M 20221342, and was granted Chinese patent due to its trait of high flocculation (Wang et al., 2024). Two *S. cerevisiae* strains were used as control strains, including a commercial strain CECA and a screened indigenous strain FBKL2.9126 (Wang et al., 2022).

### 2.2 Genotype exploration of *Schizosaccharomyces japonicus* based on microsatellite analysis

#### 2.2.1 Microsatellite loci screening and primers design of *Schizosaccharomyces japonicus*

**Figure 1:**
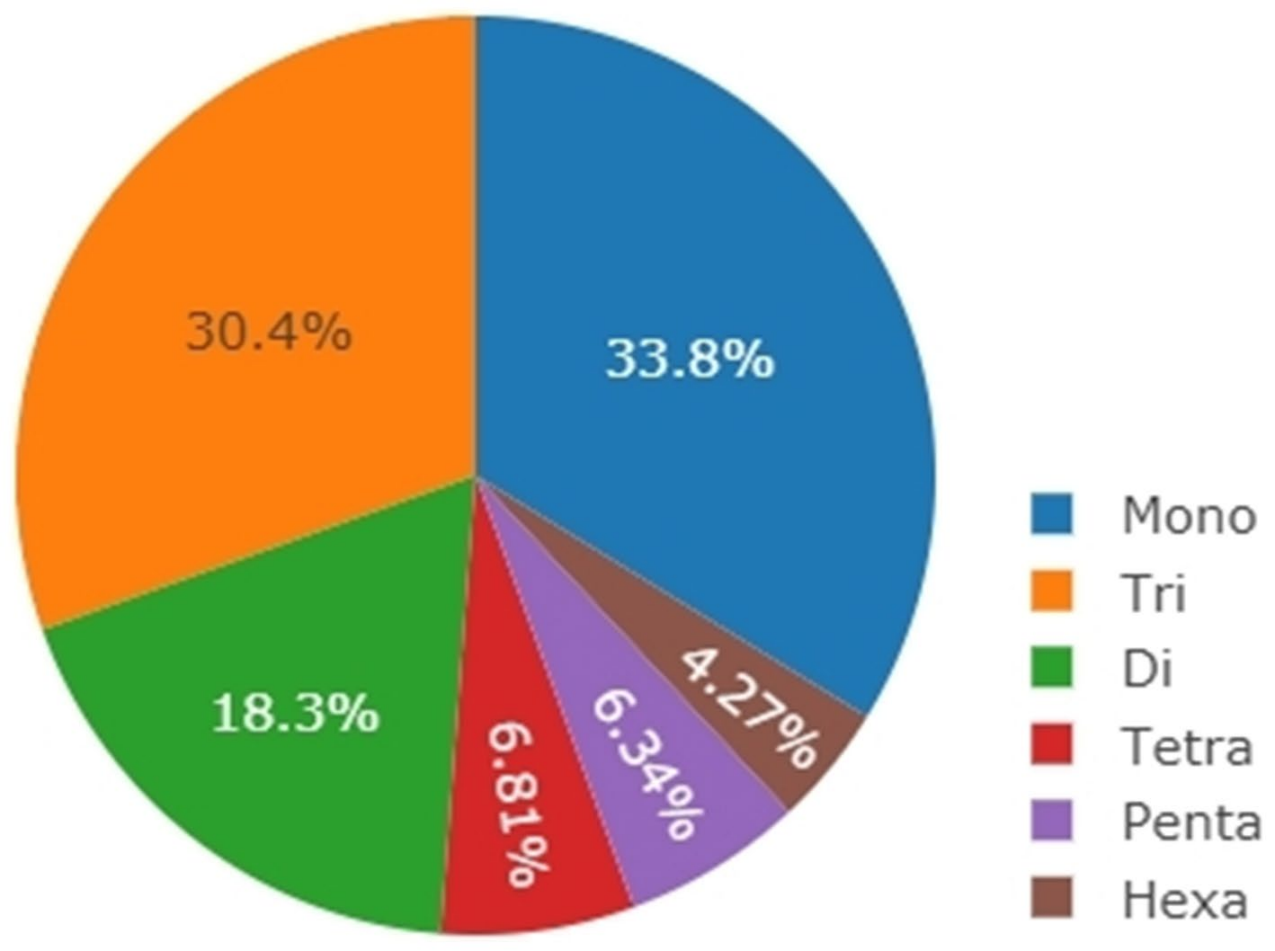
Distribution of repeat element types in the genome sequences of *S. japonicus* yFS275.

The genome sequence of *S. japonicus* strain yFS275 (NCBI sequence number NW_011627860) was used to analyze the distribution of repeat element type, including base type, number, proportion and relative abundance (Figure 1). Krait software was used to analyze microsatellite loci and to design specific primers, with the requirements of 10 base repeats of microsatellite sites, primer length between 18 bp and 20 bp, and annealing temperature between 58°C and 65°C. A total of 54 primer pairs were obtained, and 8 pairs were theoretically selected for further synthesis and confirmation, based on theoretical analysis by GenAlex 6.5 (Excel biological data analysis tool plugin) on GC content, false pairing and secondary structures probably formed. Finally, seven primer pairs were selected with high polymorphism and gene heterozygosity as shown in Table 1.

**Table 1:** The primers based on microsatellite loci of *S. japonicus*.

Locus	Primer sequence (5′–3′)	Repeat motif	Primer stability	Na	Ne	He	Ho
GA1	yFS(A)1-1F:5′-GGAGGGAGGGAATGGAAAATGT-3′ yFS(A)1-1R:5′-GCTTAAACGAAAGATGGCCCAG-3′	(GA)_13_	2.71 4.45	4	3.025	0.681	0.443
TG2	yFS(A)2-1F:5′-GCATCTGCCACGTCATATTCAC-3′ yFS(A)2-1R:5′-GCGAAGAGAAAAAGTCGTGGAG-3′	(TG)_14_	3.18 3.86	1	1	0	0
CG3	yFS(A)3-1F:5′-ATACTGCAGCAGTTTTGGCTTG-3′ yFS(A)3-1R:5′-AGCAAGCAACAAGGAAGGTAGA-3′	(CT)_14_	4.01 2.59	3	2.187	0.529	0
SaGAA1	yFS(B)1-1F:5′-AAGTTCAACGAATGCACACGAG-3′ yFS(B)1-1R:5′-ACAGCGTTATCACCATTTCCCT-3′	(GAA)_8_	4.18 4.2	2	1.051	0.044	0
SyGAA2	yFS(B)1-2F:5′-ATGACTTCCTCTGTTGACGACC-3′ yFS(B)1-2R:5′-TATCTTCGTCCACTGCTTCGTC-3′	(GAA)_9_	4.79 4.2	2	1.966	0.491	0.809
C11	yFS(C)1-1F:5′-ACTAGTACTTGGAGTTTGCGCA-3′ yFS(C)1-1R:5′-AATCCATTTGAATTGTCGGCCG-3′	(GTCT)_5_	6.09 6.13	3	2.072	0.516	0.095
C12	yFS(C)1-2F:5′-TATGGGATATGCAGTGCAACCA-3′ yFS(C)1-2R:5′-GTTCGCTTGAGCTGTCATTGTT-3′	(GTTA)_5_	3.67 2.83	2	1.433	0.255	0

Na, the number of alleles; Ne, the number of effective alleles; He, the expected heterozygosity; Ho, the observed heterozygosity.

#### 2.2.2 DNA amplification for microsatellite analysis of *Schizosaccharomyces japonicus*

The DNA amplification volume for microsatellite analysis of *S. japonicus* was 15 μL, including 2 mmol/L MgCl_2_, 1 × buffer, 0.2 mmol/L dNTPs, 0.2 μmol/L primers, 0.3 U TaqDNA polymerase, and 1 μL of DNA template. The DNA amplification procedure is predenaturation at 94°C for 5 min, 35 cycles of denaturation at 94°C for 30s, annealing at 58°C for 30s, and extension at 72°C for 30s, and final extension at 72°C for 7 min using CFX connect real-time system (Bio-Rad). The amplification products were electrophoresed in 1.5% agarose gel stained with Genegreen nucleic acid dye. The electrophoresis profiles were recorded by gel imaging system (Bio-Bset140E), and profiles of seven microsatellite loci were combined to determine the genotype of each isolate. The genotype diversity was further analyzed using chiplot.^1^

### 2.3 Metabolic capacity analysis of *Schizosaccharomyces japonicus* on malic acid and tartaric acid

Malic acid degradation solid (MDS) medium was firstly used to perform morphological observation, which contained 10 g/L yeast extract powder, 20 g/L peptone, 20 g/L glucose, 3 g/L L-malic acid, 5 g/L (NH_4_)_2_SO_4_, 0.4 g/L bromophenol blue as indicator, and 20 g/L agar. After one-week incubation at 28°C, all *S. japonicus* strains showed acid-lowering features, specifically blue-green colony, whereas *S. cerevisiae* CECA did not show acid degradation features with white colony (Supplementary Figure 1). No differences among *S. japonicus* strains could be observed from colony and microscopic cell morphology (Supplementary Figures 1, 2).

Malic acid degrading liquid (MDL) medium and tartaric acid degrading liquid (TDL) medium were further used to incubate *S. japonicus* for 7 days at 28°C in order to analyze the metabolic capacity on malic acid and tartaric acid. The only difference between MDL and TDL medium was the organic acid. The concentration of L-malic acid and tartaric acid was analyzed using enzymatic kits (Megazyme) by microplate reader FC (Thermo Fisher Scientific), and the former was observed under 340 nm and the latter under 580 nm. The standard curve of L-malic acid established in this study is *y* = 1.0643*x* + 0.0202 (*R*^2^ = 0.9949), and the standard curve of tartaric acid is *y* = 0.1173*x* + 0.0084 (*R*^2^ = 0.9942). Acid reduction rate was used to exhibit the metabolic capacity of *S. japonicus* on malic acid and tartaric acid. When the rate was between 90 and 100%, it was regarded as high metabolic capacity of acid, 60 to 90% as medium, 30 to 60% as low acid metabolic capacity.

### 2.4 The flocculence, fermentation capacity, and acid degradation analysis of *Schizosaccharomyces japonicus* in simulated fermentations

Based on the acid reduction rate and genotype, 10 *S. japonicus* strains were screened for further flocculation experiments (Table 2). In detail, *S. japonicus* strains were inoculated into YPD broth for incubation at 28°C until the stable growth stage (48 h), and then were centrifuged for 5 min at 12,000 rpm (Thermo Scientific X3R). The supernatant was removed, and cells were collected and fully mixed with deflocculation buffer. The mixture was centrifuged at 12,000 rpm for 5 min, and cells were collected and washed with sterile ddH_2_O. After removing the ddH_2_O by the same centrifuge operation, flocculation buffer was added to cells and mixed well. OD value of the cell suspension was determined at 600 nm, being recorded as A. The same cell suspension was kept at 28°C for 2 h, standing for 30 min, and the upper broth was got to determine OD value at 600 nm, being recorded as B. The flocculation value was calculated by dividing B with A, and when the value was in the range of 70 to 100%, 30 to 70%, and lower than 30%, signifying low, moderate, and high flocculence, respectively.

**Table 2:** Ten selected *S. japonicus* strains with traits of genotype, acid degradation, and flocculation.

Strain designations	Microsatellite genotypes	Malic acid degradation rate in MDL medium	Tartaric acid degradation rate in TDL medium	Flocculation value	GenBank sequence number of D1/D2 domain of 26S rRNA gene
FBKL2.9SZJ-37	28(Glb-T2a-C3b-Saa-Syc-11a-12a)	98.64%	54.49%	3.79 ± 0.08	—
FBKL2.9SZJ-6	5(Glb-T2a-C3b-Saa-Syb-11b-12a)	98.23%	62.58%	2.10 ± 0.09	—
FBKL2.9SZJ-32	23(Gla-T2a-C3a-Saa-Syc-11a-12a)	98.00%	63.57%	77.11 ± 0.46	—
FBKL2.9SZJ-34	23(Gla-T2a-C3a-Saa-Syc-11a-12a)	97.7%	61.52%	7.98 ± 0.00	—
FBKL2.9SZJ-26	19(Gla-T2a-C3c-Saa-Syc-11b-12a)	97.29%	62.30%	64.60 ± 0.17	OP364838
FBKL2.9SZJ-55	33(Gla-T2a-C3b-Saa-Syc-11a-12a)	97.27%	52.80%	10.36 ± 0.48	—
FBKL2.9SZJ-33	25(Glb-T2a-C3a-Saa-Syc-11a-12b)	97.26%	60.90%	4.84 ± 0.43	—
FBKL2.9SZJ-22	18(Gla-T2a-C3c-Saa-Syb-11b-12a)	96.85%	50.90%	10.37 ± 0.09	—
FBKL2.9SZJ-20	16(Gla-T2a-C3c-Saa-Syc-11a-12a)	96.09%	55.02%	9.53 ± 1.91	—
FBKL2.9SZJ-29	22(Glb-T2a-C3a-Saa-Syc-11b-12b)	93.52%	61.74%	5.78 ± 1.45	OP364841

—, unsequenced.

Simulated fermentations were conducted using the ten *S. japonicus* and two *S. cerevisiae* separately to analyze the fermentation capacity, and acid degradation traits of these strains during alcoholic fermentation. Two kinds of media were used for the simulated fermentations, malic acid simulated fermentation (SMF) medium and mixed acid simulated fermentation (SMTF) medium. The SMF medium contained 100 g/L glucose, 100 g/L fructose, 10 g/L yeast extract powder, 1 g/L (NH_4_)_2_SO_4_, 1 g/L MgSO_4_•7H_2_O, 3 g/L L-malic acid. 1.5 g/L malic acid and 1.5 g/L tartaric acid were used in SMTF medium. Yeast cells were inoculated at the concentration of 10^6^cells/mL into 150 mL medium in 250 mL sterile conical flask. The fermentations were performed at 28°C in triplicate, and samples were collected every 48 h to analyze the content of malic acid or tartaric acid by enzymatic kit. Fermentations were monitored by weight loss. When the weight loss was kept lower than 0.2 g/d, fermentations were regarded as termination, and the fermentation media were centrifuged at 12,000 rpm for 2 min to collect the supernatant. The ethanol concentration was measured by alcohol meter, and the residual sugar content was measured by direct titration (GB/T15038-2006, 2006).

### 2.5 Deacidification and fermentation performance analysis of screened *Schizosaccharomyces japonicus* strains in fruit wine fermentations

**Figure 2:**
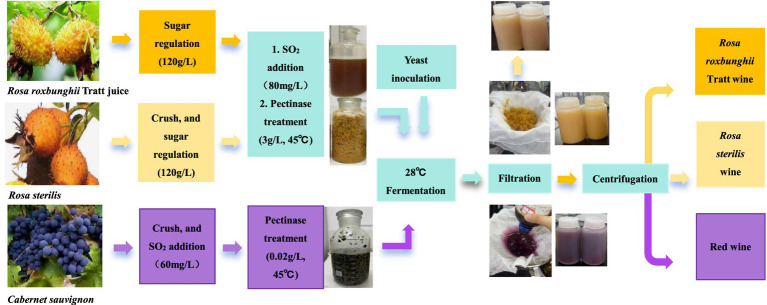
Specific fermentation treatments for three kinds of fruit.

Two *S. japonicus* strains, FBKL2.9SZJ-37 and FBKL2.9SZJ-55, were screened for further fruits wine fermentations with *S. cerevisiae* FBKL2.9126 being control strain. Three kinds of fruit were fermented including *Rosa sterilis*, *Rosa roxbunghii* Tratt, and *Cabernet Sauvignon*. *Rosa sterilis* was collected in Jintu village Liuguan town in Guizhou province (coordinate: 106.2, 26.3). *Rosa roxbunghii* Tratt juice was made from the variety of Guilong number 5 by a local company in Longli country of Guizhou Province (coordinate: 106.9, 26.5). And *Cabernet Sauvignon* was obtained from Huailai county in Hebei province (coordinate: 115.5, 40.4). All the fruits were collected in October, 2022, whereas the content of sugar and acid differed. *Rosa sterilis* contained 101.3 g/L sugar, 47.49 g/L malic acid, 1.23 g/L tartaric acid, and pH 3.27. *Rosa roxbunghii* Tratt contained 82.4 g/L sugar, 49.86 g/L malic acid, 1.46 g/L tartaric acid, and pH 3.29. *Cabernet Sauvignon* contained 229.12 g/L sugar, 2.98 g/L malic acid, 3.93 g/L tartaric acid, and pH 3.37. Fermentations were performed at 28°C in 500 mL sterile glass bottle with 350 mL juice or must and inoculum of 10^6^ cells/mL. The specific fermentation treatments were shown in Figure 2. The content of malic acid and tartaric acid was measured every 48 h using enzymatic kits, and weight loss was monitored every day. When the weight loss kept lower than 0.2 g, fermentations terminated by centrifuge at 8,000 rpm for 5 min (Beckman Coulter Allegra X-30R). The content of residual sugar, ethanol, glycerol, and pH of wines were analyzed. The content of residual sugar and ethanol was detected as described in 2.4. The pH value was determined by pH meter, the content of volatile acid was titrated by the index method (GB/T15038-2006, 2006), the glycerol content was measured by enzymatic kit (Applygen Technology).

## 3 Results

### 3.1 Genotype diversity of *Schizosaccharomyces japonicus* by screened microsatellite loci

**Figure 3:**
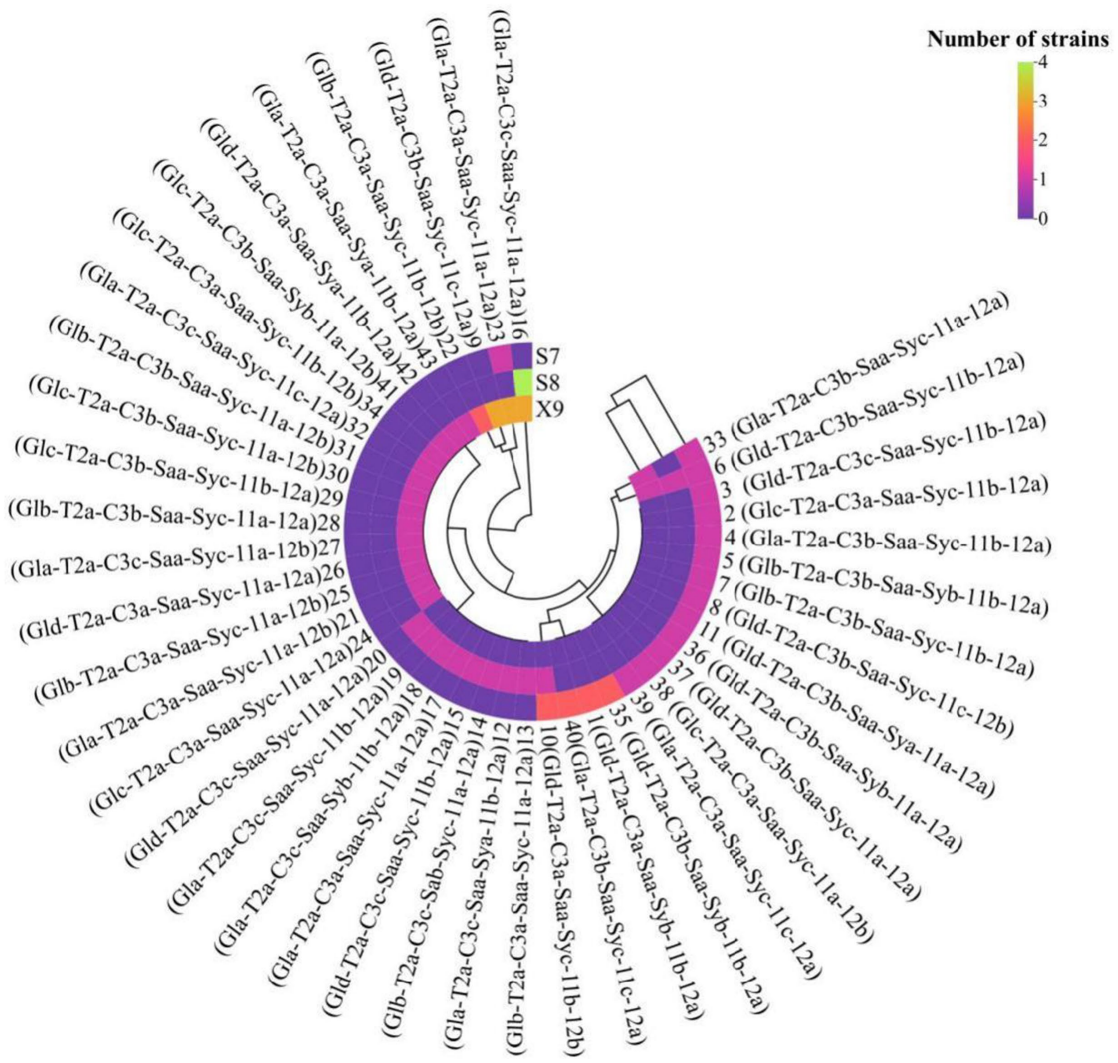
Cluster analysis of 43 genotypes of *S. japonicus* from three sources S7, S8, and X9. S7 is the abbreviation of Sandu county in July, S8 is the abbreviation of Sandu county in August, and X9 is the abbreviation of Xifeng county in September.

Forty-three genotypes were identified from 63 *S. japonicus* isolates by seven microsatellite loci (Figure 3). The seven microsatellite loci showed different discrimination ability, with locus GA1 identifying four electrophoresis profiles of Gla, Glb, Glc, Gld, being the most discriminative locus (Supplementary Figure 3). Three kinds of electrophoresis profile were separately obtained by loci CG3(C3a, C3b, C3c), SyGAA2(Sya, Syb, Syc), and C11(11a, 11b, 11c). Microsatellite loci SaGAA1(Saa, Sab) and C12(12a, 12b) identified two types of electrophoresis profile respectively, and locus TG2 only showed one type of profile T2a. The genotype 6 was the only common genotype shared by *S. japonicus* isolates from three sources. The genotype 10 was isolated from both S7 and S8, genotypes 23 and 33 were shared by the two sources S7 and X9, and the genotype 16 was shared by the two sources S8 and X9. The other 38 genotypes only appeared in one isolation source.

### 3.2 Acid-lowering features of *Schizosaccharomyces japonicus* in experimental mediums

**Figure 4:**
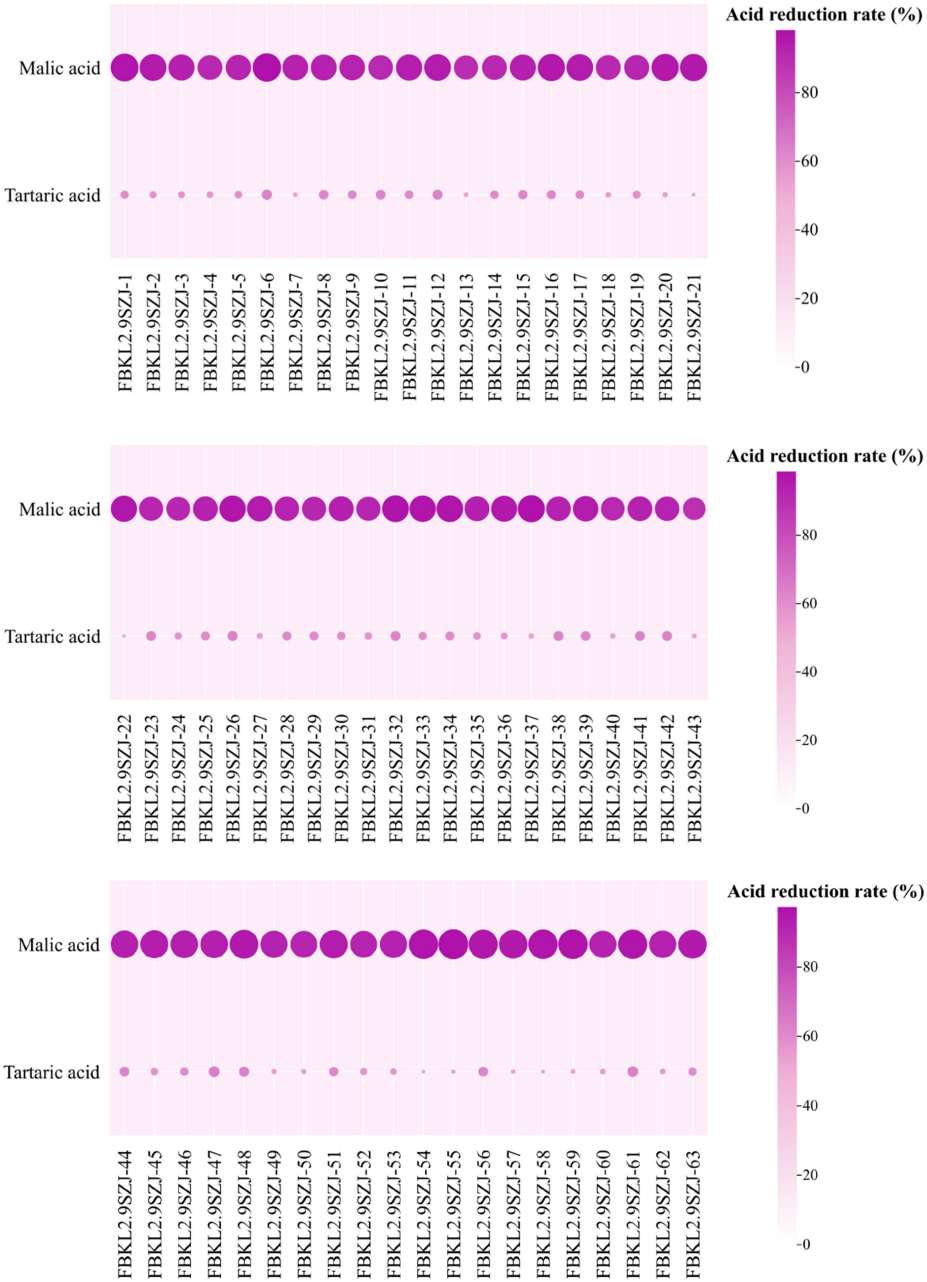
Difference of malic acid and tartaric acid metabolism capacity of 63 *S. japonicus* strains. The larger the circle, the darker the color, the stronger the ability to metabolize acid.

*S. japonicus* isolates showed relatively high malic acid reduction rate in MDL medium and low tartaric acid reduction rate in TDL medium (Figure 4). There were 62 isolates exhibiting a malic acid reduction rate higher than 90%, and were regarded as high reduction strain of malic acid. FBKL2.9SZJ-37 owned the highest malic acid reduction rate 98.64%, while the only isolate with medium metabolic capacity of malic acid, FBKL2.9SZJ-43, showed malic acid reduction rate of 89.47%. All *S. japonicus* isolates exhibited the tartaric acid reduction rate between 50.9 and 63.71%, and thus were regarded as low or medium reduction strain of tartaric acid.

### 3.3 Fermentation characteristics of screened yeast strains in simulated fermentations

Ten screened *S. japonicus* and two *S. cerevisiae* controls were used to conduct simulated fermentations separately. These strains showed different fermentation capacity in the same fermentation medium, while the same strain showed similar fermentation capacity between the two fermentation mediums SMF and SMTF (Table 3). That is, the strain used for alcoholic fermentation affected fermentation capacity more than the initial organic acid type. The 12 strains in SMF fermentation produced ethanol with content of 9.3–11.7%vol, and left 2.99–16.91 g/L residual sugar over. When they were compared with *S. cerevisiae* CECA and FBKL2.9126, two of the 10 *S. japonicus* strains produced similar ethanol with CECA, while the rest produced higher ethanol than CECA and lower ethanol than FBKL2.9126; Seven *S. japonicus* strains showed similar residual sugar content with *S. cerevisiae* FBKL2.9126, while two strains FBKL2.9SZJ-26 and FBKL2.9SZJ-29 showed relatively higher residual sugar content than *S. cerevisiae* CECA. FBKL2.9SZJ-37, FBKL2.9SZJ-34, and FBKL2.9SZJ-55 were the three *S. japonicus* strains being regarded with better fermentation capacity than other *S. japonicus* isolate due to the strong ethanol producing ability (higher than 10.5%vol) and sugar metabolism ability (residual sugar content lower than 4 g/L). The 12 strains in SMTF fermentation produced ethanol with content of 9.5–11.2%vol, and left 2.58–12.38 g/L residual sugar over. FBKL2.9SZJ-37, FBKL2.9SZJ-34, and FBKL2.9SZJ-55 were still regarded as the *S. japonicus* strains with good fermentation capacity due to similar fermentation traits in both fermentation media.

**Table 3:** Fermentation characteristics of 12 strains in simulated fermentations.

Strain designations	Ethanol (%vol)		Residual sugar (g/L)	
	SMF	SMTF	SMF	SMTF
FBKL2.9SZJ-37	10.9 ± 0.2^b^	10.9 ± 0.1^ab^	2.99 ± 2.08^ab^	6.67 ± 2.92^abc^
FBKL2.9SZJ-6	9.3 ± 0.1^d^	9.7 ± 0.6^d^	5.24 ± 3.41^ab^	4.71 ± 2.07^bc^
FBKL2.9SZJ-32	9.3 ± 0.3^d^	9.5 ± 0.4^a^	2.8 ± 1.14^a^	2.93 ± 1.93^c^
FBKL2.9SZJ-34	10.9 ± 0.1^b^	11 ± 0^ab^	2.99 ± 1.30^a^	2.58 ± 0.73^c^
FBKL2.9SZJ-26	9.4 ± 0.5^d^	9.5 ± 0.3^d^	15.09 ± 1.85^de^	12.16 ± 5.23^a^
FBKL2.9SZJ-55	10.5 ± 0.2^b^	10.4 ± 0.2^bc^	3.58 ± 0.33^ab^	6.09 ± 0.60^bc^
FBKL2.9SZJ-33	9.6 ± 0.3^cd^	9.8 ± 0.3^cd^	8.09 ± 0.80^bc^	9.89 ± 2.80^ab^
FBKL2.9SZJ-22	10.3 ± 0.6^bc^	10.4 ± 0.5^bc^	11.00 ± 3.74^cd^	10.73 ± 2^ab^
FBKL2.9SZJ-20	9.4 ± 0^d^	9.7 ± 0.2^d^	6.13 ± 1.99^ab^	3.56 ± 1.90^c^
FBKL2.9SZJ-29	9.7 ± 0.6^cd^	10 ± 0^cd^	16.91 ± 3.56^e^	12.38 ± 6.42^a^
CECA	9.3 ± 0.5^d^	9.5 ± 0.3^d^	7.09 ± 2.8^abc^	5.60 ± 2.97^bc^
FBKL2.9126	11.7 ± 0.1^a^	11.2 ± 0^a^	3.96 ± 1.9^ab^	5.38 ± 1.12^bc^

The superscript lowercase letters (a, b, c, d, and e) represented significant difference among different strains in the same fermentation mode, which was analyzed by one-way ANOVA using SPSS software (*p* < 0.05).

**Figure 5:**
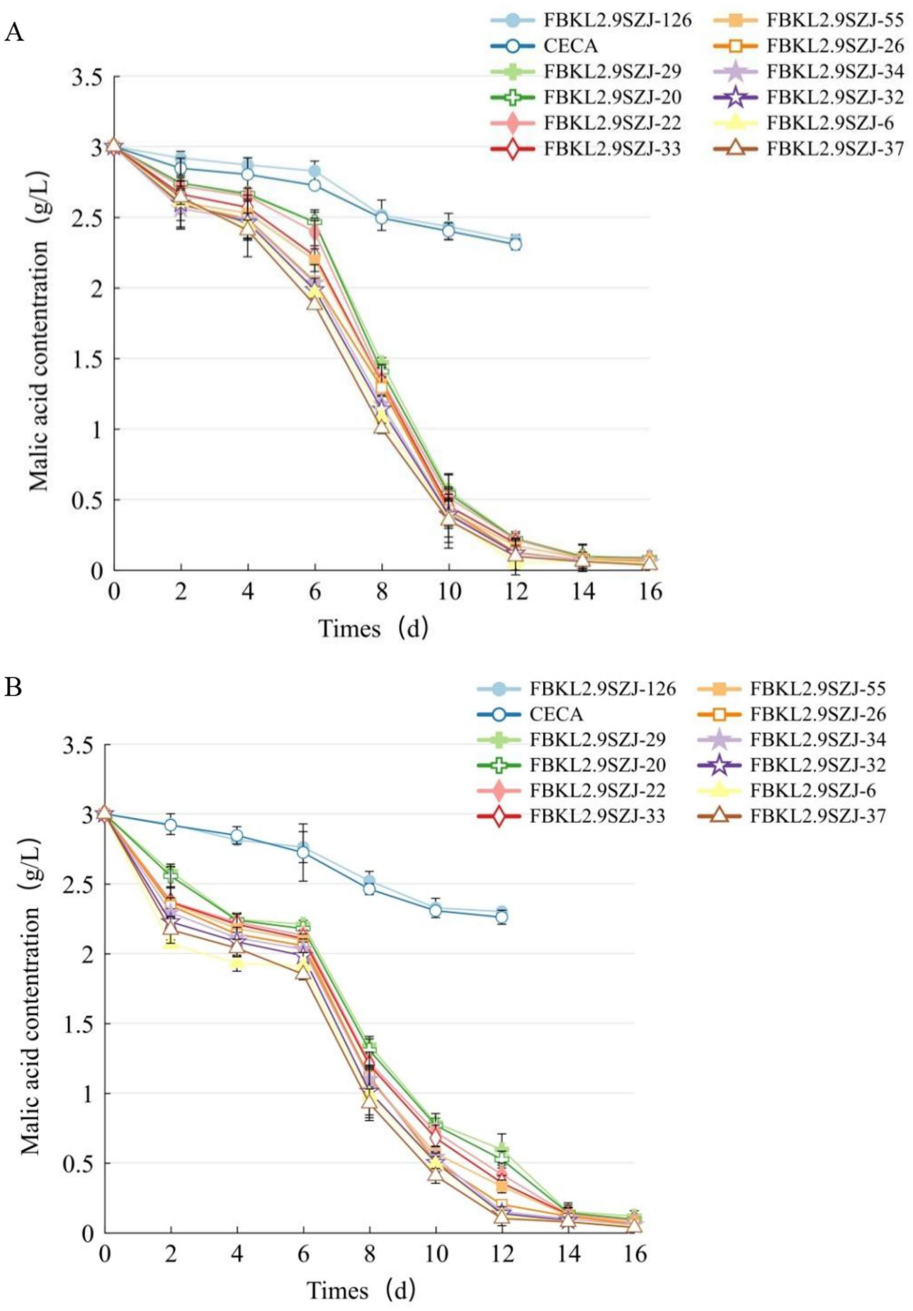
The variation of malic acid content during simulated fermentations SMF **(A)** and SMTF **(B)** inoculated with different strains.

The acid degradation traits differed among the 12 strains during alcoholic fermentation. From the perspective of malic acid degradation, *S. cerevisiae* showed a much weaker capacity than *S. japonicus*, the concentration in which at the end of fermentation (12 days) was equal to the level in *S. japonicus* at the day 6. All the 10 *S. japonicus* showed a rapid decrease of malic acid content during 6 days–10 days and a gradual decrease of malic acid content at 12 days–16 days in the two fermentation mediums SMF and SMTF (Figure 5). FBKL2.9SZJ-37 and FBKL2.9SZJ-6 owned stronger ability than the other eight *S. japonicus* strains, with the malic acid reduction rate higher than 98.5%. The initial organic acid type affected the malic acid reduction rate of the same strain, on one hand, the reduction rate was slower in SMF than in SMTF during 0 day–2 days and 10 days–12 days, on the other hand, the total reduction rate was different, for example, FBKL2.9SZJ-29 reduced 96.1 and 97.4% malic acid in SMTF and SMF separately.

### 3.4 Fermentation performance of screened strains in fruits wine fermentations

Two screened *S. japonicus* strains, FBKL2.9SZJ-37 and FBKL2.9SZJ-55, were used for further fruits wine fermentations with *S. cerevisiae* FBKL2.9126 being control strain. All inoculated yeast strains showed high viability in fruits wine fermentations, with *S. cerevisiae* keeping 10^7^ CFU/mL–10^8^ CFU/mL and *S. japonicus* being 10^6^ CFU/mL–10^7^ CFU/mL. Yeast influence on ethanol and residual sugar content was less than the influence of different fruits (Table 4). *S. cerevisiae* in *Cabernet Sauvignon* and *Rosa roxbunghii* Tratt fermentations showed slightly stronger ethanol production capacity than the two *S. japonicus* strains, but in the fermentations of *Rosa sterilis* the advantage of *S. cerevisiae* was not obvious. In each kind of fruit wine fermentation, *S. cerevisiae* FBKL2.9126 showed higher residual sugar, malic acid and tartaric acid content, and lower pH and glycerol content than the two *S. japonicus* strains. The two *S. japonicus* strains showed more similar fermentation performance than *S. cerevisiae*.

**Table 4:** The fermentation characteristics of three strains in three kinds of fruit fermentation.

Fermentation	Strain	Ethanol (%vol)	Residual sugar (g/L)	pH	Glycerol (g/L)	Malic acid (g/L)	Tartaric acid (g/L)	Volatile acid (g/L)
*Cabernet Sauvignon*	FBKL2.9SZJ-37	11 ± 0.1^b^	3.1 ± 0.06^i^	3.68 ± 0.04^a^	12.59 ± 0.12^a^	0.05 ± 0.01^g^	1.88 ± 0.01^g^	0.08 ± 0.01^e^
	FBKL2.9SZJ-55	10.8 ± 0.2^b^	3.4 ± 0.03^h^	3.66 ± 0.02^a^	12.65 ± 0.16^a^	0.09 ± 0.01^g^	1.78 ± 0.01^g^	0.09 ± 0.01^e^
	FBKL2.9126	11.7 ± 0.2^a^	4.2 ± 0.04^g^	3.45 ± 0.04^cd^	9.84 ± 0.41^b^	2.68 ± 0.01^f^	3.64 ± 0.01^f^	0.07 ± 0.01^e^
*Rosa roxbunghii* Tratt	FBKL2.9SZJ-37	5.8 ± 0.2^c^	15.4 ± 0.03^f^	3.63 ± 0.02^ab^	7.30 ± 0.08^c^	17.46 ± 0.34^e^	0.98 ± 0.02^f^	1.89 ± 0.04^a^
	FBKL2.9SZJ-55	5.4 ± 0.2^d^	16.6 ± 0.01^e^	3.59 ± 0.01^b^	7.21 ± 0.07^c^	20.79 ± 0.12^c^	0.88 ± 0.01^h^	1.62 ± 0.04^c^
	FBKL2.9126	5.7 ± 0.2^cd^	16.7 ± 0.02^d^	3.33 ± 0.04^e^	6.31 ± 0.04^d^	47.27 ± 0.08^a^	1.38 ± 0.01^d^	1.82 ± 0.02^b^
*Rosa sterilis*	FBKL2.9SZJ-37	4.3 ± 0.1^f^	18.9 ± 0.04^c^	3.51 ± 0.03^c^	6.37 ± 0.11^d^	18.76 ± 0.18^d^	0.97 ± 0.01^f^	1.76 ± 0.05^b^
	FBKL2.9SZJ-55	4.4 ± 0.1^f^	19.2 ± 0.01^b^	3.44 ± 0.03^d^	6.64 ± 0.26^d^	20.78 ± 0.31^c^	0.92 ± 0.01^g^	1.44 ± 0.04^d^
	FBKL2.9126	4.8 ± 0.1^e^	25.3 ± 0.02^a^	3.29 ± 0.04^e^	5.16 ± 0.14^e^	45.36 ± 0.09^b^	1.13 ± 0.01^e^	1.66 ± 0.04^c^

The superscript lowercase letters (a, b, c, d, e, f, g, h, and i) represented significant difference results analyzed by one-way ANOVA using SPSS software (*p* < 0.05).

The fermentation performance inoculated with the same yeast strains differed obviously due to the fruit used. The content of ethanol and glycerol in *Cabernet Sauvignon* wine were about twice of *Rosa sterilis* wine and *Rosa roxbunghii* Tratt wine, especially *Rosa sterilis* wine containing the lowest content of ethanol (4.3%vol–4.8%vol) and glycerol (5.16 g/L–6.37 g/L). Correspondingly, *Rosa sterilis* wine containing the highest residual sugar content 18.9 g/L–25.3 g/L, followed by *Rosa roxbunghii* Tratt wine and *Cabernet Sauvignon* wine. The content of malic acid and tartaric acid in each wine decreased after alcoholic fermentation, while the *Rosa sterilis wine* and *Rosa roxbunghii* Tratt wine contained higher malic acid than *Cabernet Sauvignon* wine in spite of similar pH among the three fruits wine. The volatile acid content of *Cabernet Sauvignon* wine was less than 0.1 g/L, while that of *Rosa sterilis* wine and *Rosa roxbunghii* Tratt wine was greater than 1 g/L.

**Figure 6:**
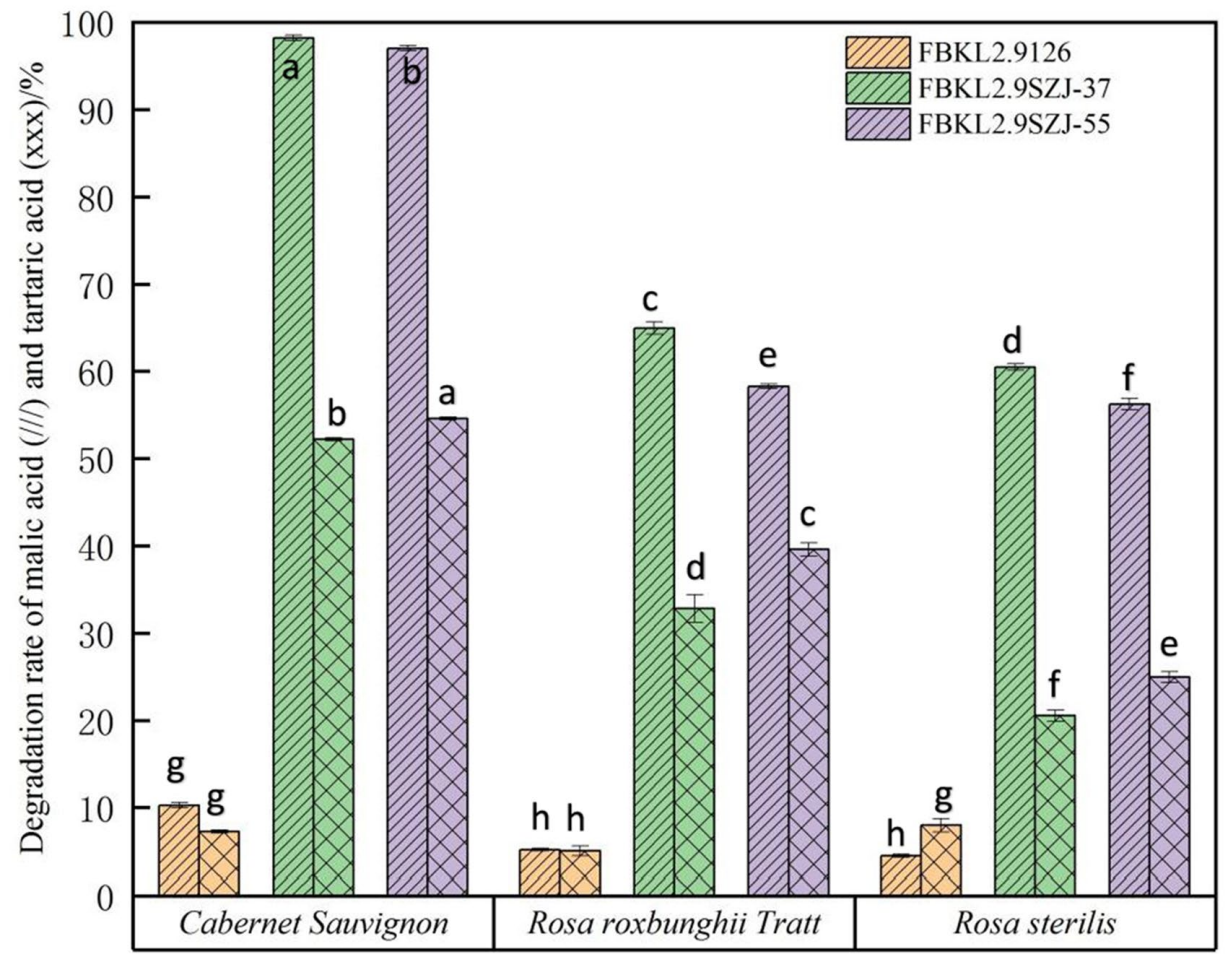
Degradation rates of malic acid and tartaric acid in three kinds of fruits fermentations inoculated with three yeast strains. The superscript lowercase letters (a, b, c, d, e, f, g, and h) represented significant difference among different strains in the same fermentation mode, which was analyzed by one-way ANOVA (*p* < 0.05).

**Figure 7:**
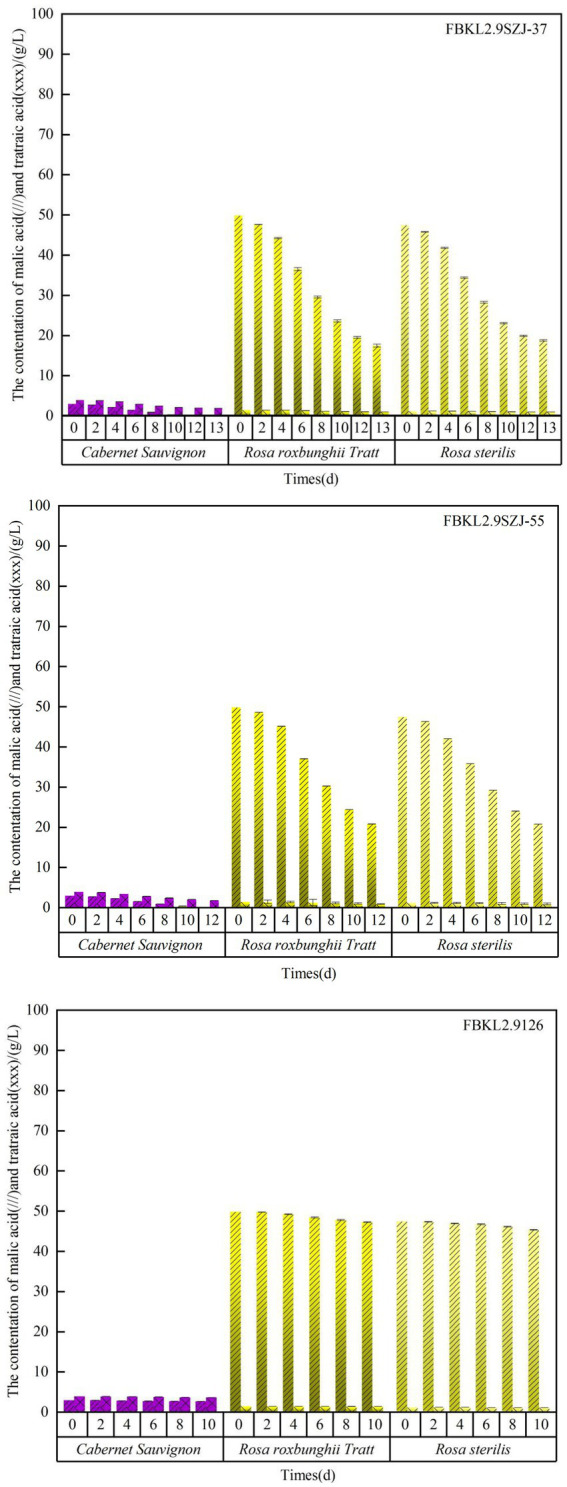
The content variation of malic acid and tartaric acid during fermentation process of three kinds of fruits inoculated with three strains.

When it comes to the deacidification performance, undoubtedly, the two *S. japonicus* strains FBKL2.9SZJ-37 and FBKL2.9SZJ-55 showed relatively higher deacidification rate than the control strain *S. cerevisiae* FBKL2.9126 (Figure 6). FBKL2.9SZJ-37 exhibited higher malic acid degradation rate and lower tartaric acid degradation rate than FBKL2.9SZJ-55 in all fruits wine fermentations. The highest malic acid degradation rate appeared in *Cabernet Sauvignon* wine fermented by FBKL2.9SZJ-37 (98.32%), and the lowest was in *Rosa sterilis* wine fermented by FBKL2.9SZJ-55 (56.24%). Correspondingly, the highest tartaric acid degradation rate appeared in *Cabernet Sauvignon* wine fermented by FBKL2.9SZJ-55 (54.71%), and the lowest was in *Rosa sterilis* wine fermented by FBKL2.9SZJ-37 (20.88%). *Rosa sterilis* and *Rosa roxbunghii* Tratt are fruits with high malic acid, and therefore the malic acid content decrease in both fruits were actually higher than *Cabernet Sauvignon* by alcoholic fermentations. The acid decreasing process of the three strains during alcoholic fermentation was shown in Figure 7. The two *S. japonicus* strains decreased malic acid faster than tartaric acid in all fruit wine fermentations. The consumption acceleration of malic acid started since the day 2, while tartaric acid being the day 4.

## 4 Discussion

Winemaking has tended from standardization and stabilization to specialization and diversification. There is growing attention to the contribution of non-*Saccharomyces* to wine quality, since non-*Saccharomyces* yeasts can enrich the flavor and taste of wine by producing secondary metabolites and extracellular enzymes, forming more complex wine characteristics (Beatriz et al., 2016). Therefore, several non-*Saccharomyces* species have been commercialized and used by winemakers to produce wines with unique flavors. Some of the commercialized non-*Saccharomyces* have relatively high fermentation capacity, but mostly were mixed with *S. cerevisiae* during fermentation to increase wine complexity (Wang et al., 2023; Portaro et al., 2022). *S. japonicus* has proved to be fermentative non-*Saccharomyces* yeast, which could coexist with *S. cerevisiae* in the late fermentation stage according to our previous studies (Wang et al., 2022). Related studies have also shown that *S. japonicus* can reduce acid and ethanol content, increase the content of glycerol, volatile compounds and active polysaccharides, and thus improving wine complexity (Domizio et al., 2017; Domizio et al., 2018; Romani et al., 2018). This study for the first time analyzed the deacidification performance of indigenous *S. japonicus* from China, and applied them to ferment *Cabernet Sauvignon* and the other two local specific fruits in Guizhou by single inoculation. The development of microsatellites loci provided method for *S. japonicus* genotype identification.

Microsatellite analysis has been regarded as one of the most reliable and stable method for genotype identification, such as the application in *S. cerevisiae* and *H. uvarum* to compare results from different laboratories (Wang et al., 2023; Yang et al., 2021). The selection of microsatellite loci is critical in the primers design for microsatellite analysis, and the repeat number of the selected core microsatellite sequences should be at a high level (Weber, 1990). Accordingly, this study developed the microsatellite loci and primers of *S. japonicus* for the first time, and applied them to analyze the genotype diversity of indigenous *S. japonicus* from Guizhou, China. *S. japonicus* from different sources showed rich genotype diversity, most genotypes were specific for their isolation sources, and several common genotypes were found from different sources, which showed the high polymorphism and gene heterozygosity of the designed primers. The genotype diversity analysis of indigenous *S. japonicus* could provide theoretical basis for further genetic study and fermentation control of *S. japonicus*.

Deacidification is a complex biochemical and winemaking process (Whiting, 1976), and several non-*Saccharomyces* yeast species have been reported to possess the ability of degrading malic acid, such as *Pichia fermentans* and *Issatchenkia terricola* (Wang et al., 2011). *S. pombe* was applied to reduce malic acid to ensure the microbial stability of wine in some warm viticulture areas in southern Spain (Benito et al., 2015). This study validated the reducing characteristics of malic acid and tartaric acid of all *S. japonicus* isolates, showing high degradation capacity of malic acid (the deacidification rate higher than 89%) and low or medium tartaric acid degradation ability (the deacidification rate less than 65%). The results demonstrated that non-*Saccharomyces* can degrade different organic acid types, the degradation mechanism is still needed to elucidate. Previous studies reported the malic acid reduction rate of *S. japonicus* was 29–83% (Romani et al., 2018; Domizio et al., 2018), and indigenous *S. japonicus* in this study showed a stronger malic acid reduction rate. FBKL2.9SZJ-37 had the strongest malic acid lowering capacity with the degradation rate higher than 98% in both simulated medium MDL and *Cabernet Sauvignon* fermentation. Higher than 60% malic acid in both *Rosa roxbunghii* Tratt (32.4 g/L) and *Rosa sterilis* (28.73 g/L) were degraded during the alcoholic fermentation of FBKL2.9SZJ-37, with the *S. cerevisiae* control being 4 to 5%. Therefore, the screened *S. japonicus* with excellent deacidification trait is potential for improving the harsh and sharp sour taste of the malic acid type fruit such as *Rosa roxbunghii* Tratt and *Rosa sterilis*.

Besides deacidification traits, the two screened *S. japonicus* strains, FBKL2.9SZJ-37 and FBKL2.9SZJ-55, showed good fermentative traits in three kinds of fruits wine fermentations. In detail, when compared to the excellent indigenous *S. cerevisiae* FBKL2.9126 (Wang et al., 2022), the two *S. japonicus* strains showed similar ethanol production ability, stronger sugar consumption and glycerol production ability. In detail, almost 1 g/L higher glycerol were produced by *S. japonicus* in both *Rosa roxbunghii* Tratt and *Rosa sterilis* wines than the *S. cerevisiae* FBKL2.9126 with high glycerol production trait (Wang et al., 2022), and in *Cabernet Sauvignon* wine being more (higher than 2.5 g/L). The stronger glycerol production trait of *S. japonicus* enables further taste improvement by the balance between the round and harsh brought by glycerol and malic acid subjectively. However, the influence of yeast strain on wine difference was less than the influence of different fruits used due to the quite different sugar and acid content among *Cabernet Sauvignon*, *Rosa sterilis*, and *Rosa roxbunghii* Tratt. When the same yeast strain was inoculated into different fruits, the content of ethanol and glycerol in *Cabernet Sauvignon* wine was about twice of *Rosa sterilis* wine and *Rosa roxbunghii* Tratt wine, even *S. cerevisiae* being used. The phenomenon illustrated the nutrient limitation of *Rosa sterilis* and *Rosa roxbunghii* Tratt on alcoholic fermentation, which still need further investigation. In addition, the *Rosa sterilis* wine and *Rosa roxbunghii* Tratt wine contained higher malic acid and volatile acid than *Cabernet Sauvignon* wine in spite of similar pH among the three fruits wine. Furthermore, the indigenous *S. japonicus* showed different flocculation traits, which probably provide convenience for wine clarification process (Wang et al., 2024). Combined with various characteristics of *S. japonicus*, it is beneficial to promote the further development of indigenous *S. japonicus* resources.

In conclusion, this study developed microsatellite loci and primers for *S. japonicus*, evaluated their genotype diversity, and found common genotype from different isolation sources. The traits of flocculation, deacidification, and fermentation were further investigated in this study, and strain FBKL2.9SZJ-37, which owned the specific genotype 28 from source X9, had the most prominent ability of depredating malic acid. As a dominant non-*Saccharomyces* yeast species found in the late stage of natural fermentations, further studies on *S. japonicus* can promote the development and commercialization of excellent indigenous non-*Saccharomyces* yeast resources. Especially for the special fruit wine including *Rosa roxbunghii* Tratt and *Rosa sterilis* wine, the “easy to drink” type with low ethanol and malic acid has been increasingly concerned by the youngsters, which demands for specific commercial yeast species developed.

## Data Availability

The datasets presented in this study can be found in online repositories. The names of the repository/repositories and accession number(s) can be found in the article/Supplementary material.

## Footnotes

^1^ https://www.chiplot.online/

## References

[ref1] An H. Liu M. Yang M. Fan W. (2011). Analysis of organic acid components and ascorbate content in roxburgh rose. Agric. Sci. China 44, 2094–2100. doi: 10.3846/j.issn.0578-175.2011.10.014

[ref2] Beatriz P. Gil J. V. Paloma M. (2016). Past and future of non-*Saccharomyces* yeasts: from spoilage microorganisms to biotechnological tools for improving wine aroma complexity. Front. Microbiol. 7:411. doi: 10.3389/fmicb.2016.00411 27065975 PMC4814449

[ref3] Benito Á. Calderón F. Benito S. (2016). Combined use of *S. pombe* and *L. thermotolerans* in winemaking. Beneficial effects determined through the study of wines’ analytical characteristics. Molecules 21:1744. doi: 10.3390/molecules21121744 27999345 PMC6273388

[ref4] Benito Á. Calderón F. Palomero F. Benito S. (2015). Combine use of selected *Schizosaccharomyces pombe* and *Lachancea thermotolerans* yeast strains as an alternative to the traditional malolactic fermentation in red wine production. Molecules 20, 9510–9523. doi: 10.3390/molecules20069510 26016543 PMC6272599

[ref5] Cao Y. Geng Y. Han N. Wang Z. Wang X. Sun Z. (2023). Research on organic acids and acid reduction strategies in fruit wine. Food Ind. Sci. Technol. 44, 457–464. doi: 10.13386/j.issn1002-0306.2022090119

[ref6] Chen Q. Xie H. He J. Wu F. Wang C. (2024). Evaluation of the brewing characteristics of five characteristic fruits in Guizhou based on the composition of organic acids and polyphenols. Food Sci. 45, 10158–10166. doi: 10.7506/spkx1002-6630-20230715-176

[ref7] Ding Z. Sun Y. (2024). Enhancing the taste of an unsung fruit. Nature Available online at: https://www.nature.com/articles/d42473-024-00137-9

[ref8] Domizio P. Lencioni L. Calamai C. Portaro L. Bisson L. (2018). Evaluation of the yeast *Schizosaccharomyces japonicus* for use in wine production. Am. J. Enol. Vitic. 69, 266–277. doi: 10.5344/ajev.2018.18004

[ref9] Domizio P. Liu Y. Bisson L. F. Barile D. (2017). Cell wall polysaccharides released during the alcoholic fermentation by *Schizosaccharomyces pombe* and *S. japonicus*: quantification and characterization. Food Microbiol. 61, 136–149. doi: 10.1016/j.fm.2016.08.010, PMID: 27697163 PMC5113737

[ref10] Franco-Duarte R. Umek L. Zupan B. Schuller D. (2009). Computational approaches for the genetic and phenotypic characterization of a *S. cerevisiae* wine yeast collection. Yeast 26, 675–692. doi: 10.1002/yea.1728 19894212

[ref11] GB/T15038-2006 (2006). Analytical methods of wine and fruit wine. Beijing: China Standard Press.

[ref12] Masneuf-Pomarede I. Bely M. Marullo P. Albertin W. (2016). The genetics of non-conventional wine yeasts: current knowledge and future challenges. Front. Microbiol. 6:1563. doi: 10.3389/fmicb.2015.01563, PMID: 26793188 PMC4707289

[ref13] Pfliegler W. P. Horváth E. Kállai Z. Sipiczki M. (2014). Diversity of *Candida zemplinina* isolates inferred from RAPD, micro/minisatellite and physiological analysis. Microbiol. Res. 169, 402–410. doi: 10.1016/j.micres.2013.09.006, PMID: 24176816

[ref14] Picariello L. Rinaldi A. Martino F. Petracca F. Moio L. Gambuti A. (2019). Modification of the organic acid profile of grapes due to climate changes alters the stability of red wine pH phenolics during controlled oxidation. Vitis 58, 127–133. doi: 10.5073/vitis.2019.58.special-issue.127-133

[ref15] Portaro L. Maioli F. Canuti V. Picchi M. Lencioni L. Mannazzu I.. (2022). *Schizosaccharomyces japonicus*/*Saccharomyces cerevisiae* mixed starter cultures: new perspectives for the improvement of Sangiovese aroma, taste, and color stability. LWT 156:113009. doi: 10.1016/j.lwt.2021.113009

[ref16] Romani C. Lencioni L. Gobbi M. Mannazzu I. Ciani M. Domizio P. (2018). *Schizosaccharomyces japonicus*: a polysaccharide-overproducing yeast to be used in winemaking. Fermentation 4:14. doi: 10.3390/fermentation4010014

[ref17] Schuller D. Casal M. (2007). The genetic structure of fermentative vineyard-associated *Saccharomyces cerevisiae* populations revealed by microsatellite analysis. Antonie Van Leeuwenhoek 91, 137–150. doi: 10.1007/s10482-006-9104-8 17094015

[ref18] Shan Y. Liao X. Li B. Ding Z. Sun Y. (2024). Healthy people and a healthy planet. Nature Available online at: https://www.nature.com/articles/d42473-024-00132-0

[ref19] Volschenk H. van H. Viljoen–Bloom M. (2003). Malo-ethanolic fermentation in *Saccharomyces* and *Schizosaccharomyces*. Curr. Genet. 43, 379–391. doi: 10.1007/s00294-003-0411-6 12802505

[ref20] Wang C. Liang S. Yang J. Wu C. Qiu S. (2022). The impact of indigenous *Saccharomyces cerevisiae* and *Schizosaccharomyces japonicus* on typicality of crystal grape (Niagara) wine. Food Res. Int. 159:111580. doi: 10.1016/j.foodres.2022.111580 35940784

[ref21] Wang C. Wu C. Qiu S. (2019). Yeast diversity investigation of *Vitis davidii* Föex during spontaneous fermentations using culture-dependent and high-throughput sequencing approaches. Food Res. Int. 126:108582. doi: 10.1016/j.foodres.2019.108582, PMID: 31732056

[ref22] Wang C. Yu J. Zhou W. Xu Y. (2023). Progress in wine fermentation applications of non-*Saccharomyces*. Agric. Sci. China 56, 529–548. doi: 10.3864/j.issn.0578-1752.2023.03.011

[ref23] Wang C. Zhang Z. He J. (2024). A *Schizosaccharomyces japonicus* strain with high flocculation trait and its application. Patent number ZL202211302564.1

[ref24] Wang L. Zhang W. Wen L. (2011). Screening and identification of degradable L-malic acid and citric acid strains. Food Sci. 31, 279–282. doi: 10.7506/spkx1002-6630-201021063

[ref25] Weber J. L. (1990). Informativeness of human (dC − dA)*_n_*·(dG − dT)*_n_* polymorphisms. J. Genet. Genomics 7, 524–530. doi: 10.1016/0888-7543(90)90195-Z 1974878

[ref26] Whiting G. C. (1976). Organic acid metabolism of yeasts during fermentation of alcoholic beverages—a review. J. Inst. Brew. 82, 84–92. doi: 10.1002/j.2050-0416.1976.tb03731.x

[ref27] Yang C. Liu Y. Ge Q. (2016). Analysis of organic acid content in wine grapes at the eastern foot of Helan Mountain. Food Technol. 41, 244–247. doi: 10.13684/j.cnki.spkj.2016.11.052

[ref28] Yang J. Wu C. Wang C. Tian J. Xu Y. Qiu S. (2021). Analysis of genotype diversity of wild *S. cerevisiae* in natural fermentation of Chinese thorn grape in Ziyun, Guizhou. J. Microbiol. 61, 3431–3443. doi: 10.13343/j.cnki.wsxb.20210004

