# Supplementary material for: Targeting indigenous Schizosaccharomyces japonicus for genotype exploration and organic acid degradation analysis
Source: Front Microbiol. 2025 Jun 4;16:1569585. doi: 10.3389/fmicb.2025.1569585 (PMC12174398; doi:10.3389/fmicb.2025.1569585)
Supplement: Supplementary file 1 [file Data_Sheet_1.DOCX]

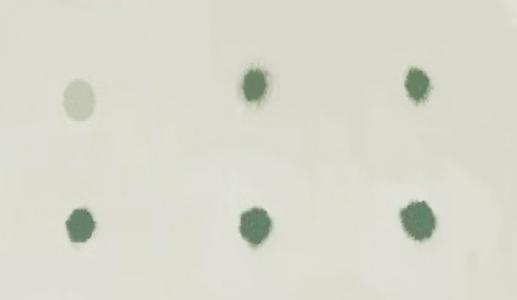


CECA

Supplementary Figure 1. Colony morphology of *S. cerevisiae* CECA and five strains of *S. japonicus* on MDS medium


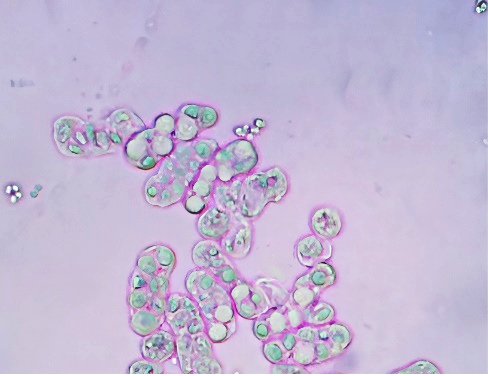

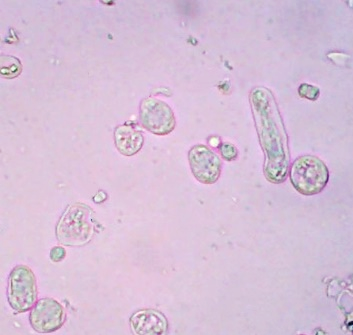


Supplementary Figure 2 *S. japonicus* cells from blue-green colony on MDS medium under optical microscope with 400 times of magnification.

| GA1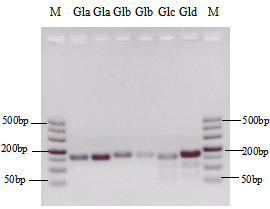 | TG2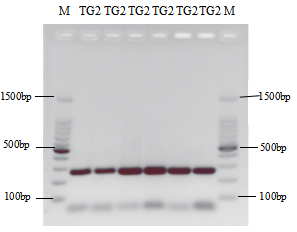 |
| --- | --- |
| CG3  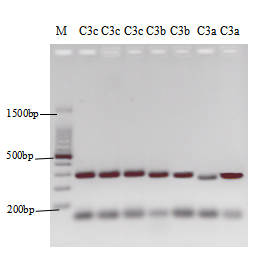 | **SaGAA1**  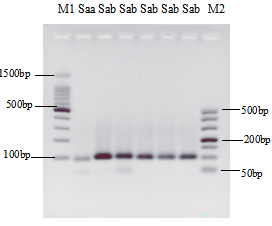 |
| SyGAA2  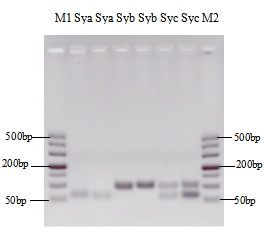 | **C11**  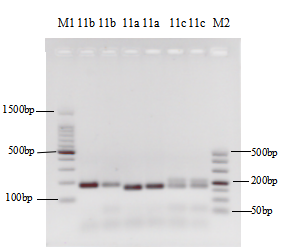 |
| C12  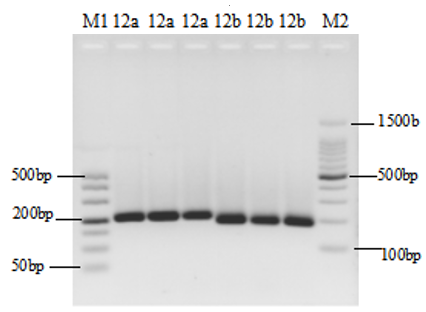 |  |

Supplementary Figure 3. Representative electrophoresis profiles of DNA amplification using seven microsatellite loci.
